# Third- and concerned-party positions in moral dilemmas: Effect of empathy on ethical judgment

**DOI:** 10.1371/journal.pone.0354169

**Published:** 2026-07-30

**Authors:** Hiroshi Nonami, Shoji Ohtomo, Toshiaki Aoki, Motoaki Sugiura, Go Sakamoto, Kentaro Oba, Yutaka Tashiro

**Affiliations:** 1 School of Sociology, Kwnsei-Gakuin University, Nishinomiya, Hyogo, Japan; 2 Faculty of Policy Studies, Chuo University, Hachioji, Tokyo, Japan; 3 Graduate school of International Cultural Studies, Tohoku University, Sendai, Miyagi, Japan; 4 Institute of Development, Aging and Cancer, Tohoku University, Sendai, Miyagi, Japan; 5 School of International Relations, University of Shizuoka, Shizuoka, Shizuoka, Japan; 6 Smart Aging Research Center, Tohoku University, Sendai, Miyagi, Japan; 7 Department of Tourism, Takarazuka University of Medical and Health Care, Miyakojima, Okinawa, Japan; University of Malta, MALTA

## Abstract

The present study proposes a categorization of moral dilemmas into third-party dilemmas in which a judge’s choice affects only the life or death of others, and concerned-party dilemmas in which not only others’ but also the judge’s life or death is altered. While the former requires the judge to make only moral judgments to save others, another rational decision-making process that considers the judge’s life and death may also impact the latter. We examined ethical judgments based on these two types of dilemmas with a focus on empathic concern. Participants in Experiment 1 were more ethically permissive to save the many including him/herself at the expense of one person in the concerned-party dilemma than in the third-party dilemma, but empathic concern in both types of dilemmas consistently suppressed such utilitarian choices derived from “instrumental harm” (IH), which permits sacrificing a few necessary when it saves many. In Experiment 2, we also confirmed the suppressive effect of empathic concern for utilitarian choices derived from IH in the Pareto concerned-party dilemma in which the death of one person remains fixed regardless of the judge’s choice. We discuss the importance of categorizing these two types of dilemmas in moral dilemmas and demonstrates the public goods dilemma as a Pareto concerned-party dilemma in everyday life.

## Introduction

Many recent neuroscientific or psychological studies clearly support the notion that emotional processes can influence human moral judgment [1–3]. They propose a dual-process model of moral judgment [4–9]. According to this model, moral judgment includes two types of processes: controllable cognitive or automatic affective. Specifically, when people need to decide whether to sacrifice one person to save lives of many, the controllable process drives people to choose to save many at the expense of one, whereas the affective process leads them to save one even at the expense of many (i.e., to choose not to harm one).

Whether controllable or affective processes are activated more predominantly (i.e., whether people adopt a choice that saves many people [utilitarian choice] or one person [nonutilitarian choice]), depends on the directness of a harm that a judge making a choice inflicts on others [10,11], the tradeoff structure of the choice-making condition [12], the judge’s self-involvement in the consequences of the choice [13], and an individual’s deliberative disposition [14]. In particular, the distinction between impersonal and personal dilemmas relative to the directness of the harm has received widespread attention since its introduction by Greene et al. [5]. In impersonal dilemmas (e.g., whether or not to pull the lever to change the path of a runaway trolley from five people to one), where the harm to one person in exchange for saving the many does not involve direct physical contact and thus less emotional aversion to sacrificing that one person, people are more likely to permit a utilitarian choice. However, in a personal dilemma in which people must directly harm one person through physical contact (e.g., whether or not to push one person off a footbridge to derail a runaway trolley headed for five people), people do not permit harm due to the high level of affective aversion, which results in an increased nonutilitarian choice.

Previous studies posed numerous scenarios of impersonal or personal dilemmas [5,11,15]. Nevertheless, others questioned the categorization of these scenarios only by one criterion: impersonal or personal. For example, Huebner et al. [12] reanalyzed impersonal or personal scenarios in a previous study by Greene et al. [11] and pointed out that it depicted emergencies or non-emergencies, killing or only slightly injuring one person. The study also highlighted that it depicted non-Pareto scenarios in which one person can be saved by sacrificing many, or Pareto scenarios in which the death of one person is unavoidable in any case (i.e., the choice to avoid the death of one person results in the death of everyone, including the one and the many) [12]. Furthermore, Moore et al. [13] compared scenarios in which the judge’s choice determined his/her life or death with other scenarios in which his/her choice affected only the life or death of others in impersonal and personal contexts. Similar to the former type of scenario, a moral dilemma in which the judge’s choice determines his/her life or death is called a self-dilemma. Judges were more permissive of the choice to save the many, including themselves, in scenarios classified under self-dilemma compared with those that depict only others’ life or death—even in the same impersonal scenarios [13]. An example of scenarios under self-dilemma presented by previous studies included villagers hiding in a cellar on the battlefield and deciding whether or not to suffocate a crying baby by holding its mouth to prevent an enemy from finding it and killing everyone, including the judge. Another is whether or not to push one seriously injured passenger overboard to save the lives of many passengers, including the judge, on a lifeboat that is imminently sinking due to overcapacity. By contrast, runaway trolleys and footbridge dilemmas are scenarios in which the judge’s choices will determine only the life or death of others.

Indeed, Greene et al. [5,11] did not distinguish between situations in which the judge’s choice does not affect his/her life or death and the judge’s life or death is altered in moral dilemmas (e.g., the footbridge and lifeboat dilemmas are categorized as personal dilemmas, although only the latter involves the life or death of the judge). However, both types must be regarded as mutually disparate in terms of the possibility of intervention through a rational decision-making process in which the judge considers personal interest. Moore et al. [13,16] argued that the distinction between the self and others, whereby killing to save oneself and others is more permissible than killing to save only others, is an evolutionary, automatic process. Although examining the automatic process is outside the scope of the current study, assuming that people faced with a self-dilemma are more likely to make choices in line with the maximin principle of avoiding one’s maximum disadvantage given a set of options is reasonable. In other words, we can assume that utilitarian choices that lead to the self-preservation of the judge are more permissive in a self-dilemma in which the judge’s choice affects the life or death of others, including his/her own, compared with a dilemma in which his/her choice determines only the life or death of others [12,13].

The first objective of the current study is to propose a categorization of moral dilemmas in which the judge’s choice affects/does not affect his/her life or death. In the present study, the former and latter scenarios are referred to as the concerned- and third-party dilemmas, respectively [17]. Just as people’s choices in the trolley problem vary according to their relationship with hypothetical victims, one can infer that several everyday moral dilemmas that involve costs and benefits for the judges, indeed, occur [13,16]. Establishing the categorization of third- or concerned-party dilemmas is an important insight into the prediction of decisions and choices people make when faced with real moral dilemmas in everyday familiar places, instead of vignette-based scenarios.

The second objective is to examine the contributions of affective processes to moral judgment in the contexts of third- and concerned-party dilemmas. The present study focuses on the relationship between moral judgment and empathic concern, defined as the tendency to evoke feelings of compassion and concern for others [18,19]. Gleichgerrcht and Young [20] reported that individuals who make utilitarian choices in personal scenarios exhibit less empathic concern. In detail, they compared the trolley scenario with the footbridge scenario in the first experiment and also compared the crying baby scenario with the toxic fumes scenario (toxic fumes are headed for a hospital room with three patients; the dilemma is whether to turn on the button to redirect the path of the fumes to a room with one patient to save them) in the second. The authors found that individuals making utilitarian choices displayed less empathic concern than those making nonutilitarian choices only in footbridge and crying baby (personal) scenarios. Interpreting their report based on the abovementioned categorization, the footbridge and crying baby scenarios are third- and concerned-party dilemmas, respectively, despite being personal scenarios. Accordingly, one can assume that individuals making utilitarian choices in a personal scenario would exhibit less empathetic concern in both the third- and concerned-party dilemmas.

Furthermore, in their second experiment (comparing between the toxic fumes [impersonal/third-party] and crying baby [personal/concerned-party] scenarios), the latter exhibited a lower percentage of utilitarian choices (killing the baby; 16%) than the former (62%), although it is a concerned-party dilemma in which the judges’ life or death depends on his/her choice. Gleichgerrcht and Young [20] interpret this result in terms of impersonal/personal factors, attributing it to the former being an impersonal scenario and the latter a personal one. However, the toxic fumes and crying baby scenarios they examined differ not only in the context of impersonal versus personal but also in that the former constitutes a third-party dilemma while the latter involves a concerned-party dilemma. Based on their results, nonetheless, one can hypothesize that utilitarian choices at the expense of the few would be suppressed in personal scenarios compared with impersonal scenarios even in concerned-party dilemmas, in which the interests of the many (including the judge) and a few others, are in conflict. Comparing the trolley and footbridge scenarios as a third-party dilemma, the utilitarian choice is suppressed in the latter, which is categorized as a personal scenario [5,11]. A potential reason is the emotional aversion that emerges in response to direct harm to others; a similar response may be observed in concerned-party dilemmas. To examine the abovementioned hypothesis more rigorously, manipulating the impersonal/personal and third-/concerned-party factors independently is necessary instead of directly comparing toxic fumes (impersonal/third-party) and crying baby (personal/concerned-party) as done by Gleichgerrcht and Young (2013).

As previously noted, the third- and concerned-party dilemmas exhibit dissimilar structures in one respect: whether or not the interest of the judge is involved. The latter leaves room for the judge to consider his/her interests in accordance with the maximin principle, whereas the former does not. Hence, the latter is more likely to make utilitarian choices at the expense of the few compared with the former. Alternatively, the structure of the dilemma as a moral one, in which the justice of the choice to sacrifice the few to save the many does not differ between the third- or concerned-party dilemma. Therefore, the involvement of controllable or emotional processes leading to a utilitarian or nonutilitarian choice should be common to both types of dilemma. Hence, it is predicted that previous findings on the reduction of utilitarian choice in personal scenario compared to impersonal scenarios [4,5,10,11] and regarding the lower empathic concern of people making utilitarian choices in personal scenarios [20] would be common to the third- and concerned-party dilemmas.

The following important points must be highlighted. Most insights theoretically substantiating predictions in the present paper regarding utilitarian choices or judgments were gleaned using a dichotomous scale (“right” and “wrong”) asking whether it is ethically right to sacrifice one person to save many [5,8,11–13,20]. Such scales successfully capture the utilitarian idea of “instrumental harm” (IH), the permission required to sacrifice one person to save many [21,22]. Utilitarianism also includes the concept of “impartial beneficence” (IB), in which “[e]ach is to count for one and none for more than one” [23], which is believed would maximize the well-being of all sentient beings on the planet without privileging certain individuals over others [21,22]. This study follows previous research in adopting a dichotomous scale [5,11,13,16,20] to examine the above-mentioned predictions based on existing insights. It focuses on the concept of IH within utilitarianism. Hereafter, when “utilitarian choices” are mentioned, they should be taken as specifically indicating “utilitarian choices derived from IH.”

We first examine the following hypotheses in Experiment 1 based on this discussion. While this study did not undergo pre-registration, all hypotheses were predicated on an overview of previously derived theoretical insights and developed prior to conducting experiments. All manipulations in the experiments and subsequent analyses were formulated on the basis of the following hypotheses.

Hypothesis 1a: Ethical judgment on a utilitarian choice derived from IH would be more permissive in concerned-party dilemmas than third-party dilemmas.

Hypothesis 1b: Permission for utilitarian choice derived from IH would be reduced in personal scenarios relative to impersonal scenarios in either the third- or concerned-party dilemma.

Hypothesis 1c: In both dilemmas of third- and concerned-party dilemmas, individuals that permit utilitarian choice derived from IH in personal scenarios would exhibit less empathic concern than those that do not permit it.

Hypothesis 1a provides a re-examination of the empirical insight that in self-dilemmas (where a judge’s choice affects not only others’ lives but also his/her own), choices leading to the saving of the judge are more likely to emerge than in dilemmas where the judge’s choice affects only the lives of others [13], based on a systematic factorial design. Hypothesis 1b reexamines the findings of Greene et al. [5]—namely, that utilitarian choices are more likely to occur in impersonal than in personal dilemmas—by incorporating the categorization criteria for third- and concerned-party dilemmas.

## Experiment 1

### Participants

Among monitors registered with a private research company, participants whose native language was Japanese and who were physically and mentally healthy and aged more than 18 years were directed to a web-based questionnaire. Data collection began on 12 June 2024 and was completed within one week, on 18 June 2024. Participants gave a written electronic informed consent online in a web-based form before enrollment, and this was safely recorded as part of the submission process. The total number of respondents was *n* = 1,300. The number and demographics of valid respondents are detailed later, at the beginning of Results section.

### Materials and methods

Using a two-factorial design (both within-participant factors): directness of harm (2: impersonal or personal) × judge’s self-involvement (2: third- or concerned-party) in moral dilemmas, we prepared four types of vignettes, namely, trolley, footbridge, department store, and cemetery. Although they include the trolley and footbridge scenarios, which previous studies have repeatedly presented [5,11,15], we modified them to ensure that the four scenarios retain their contextual and consequential equivalence (see the S1 Appendix for all scenarios). They were standardized to render the number of deaths equal (i.e., one person killed to save five), while the others to be saved (or killed) were described as strangers to the participants. All vignettes consisted of 4–6 sentences and 185–194 Japanese characters (88–98 words in English). In the third-party dilemma, the participant as judge neither belongs to the five nor one, and the choice to save either one does not affect the judge’s life or death. Conversely, the concerned-party dilemma depicts the judge as one of the five sides, thus putting him/herself in a position in which his/her life or death depends on their own choices.

The trolley scenario (impersonal/third-party) consists of a utilitarian choice derived from IH to pull the lever to direct a runaway trolley from five persons to one and a nonutilitarian choice not to intervene in the trolley’s direction, which will collide with the five persons. The footbridge scenario (personal/third-party) involves a utilitarian choice derived from IH to push one person off a footbridge and derail the trolley with his body, thus preventing collision and the death of the five people on the tracks, and a nonutilitarian choice not to intervene in the trolley’s rush. These two scenarios are defined as third-party dilemmas, because the judge’s utilitarian or nonutilitarian choices does not affect his/her interests (life or death). In the department store scenario (impersonal/concerned-party), the utilitarian choice derived from IH is to turn on the ventilation switch in a department store with toxic fumes from the fire, which would save five persons, including the judge, while toxic fumes were directed to one person on another floor. The nonutilitarian choice is not to turn on the ventilation switch (as a result, five individuals, including the judge, will die from the toxic fumes). The utilitarian choice derived from IH in the cemetery scenario (personal/concerned-party) is for the judge, who stepped into a local clan cemetery while hiking, to stab one hiker to death using the clan’s sword to avoid the death of five people, including the judge. The nonutilitarian choice is not to stab one hiker to death (as a result, the clan will kill five persons, including the judge). The department store and cemetery scenarios can be viewed as concerned-party dilemmas, because the life or death of the judge is dependent on the judge’s choices. Additionally, the impersonal scenarios (trolley and department store) depict a choice in which saving five persons indirectly harms one; thus, emotional conflict is less likely to occur for the judge. Conversely, the personal scenarios (footbridge and cemetery) indicate that one person is intentionally and directly killed to save five persons, and emotional conflict is significant for the judge.

### Procedures

The participants were randomly presented with the four abovementioned scenarios. In all scenarios, they made ethical judgments on the utilitarian choice derived from IH to sacrifice one person to save five persons. They then responded to the following questions and, finally, to their personal attributes. These series of tasks are summarized as follows.

Ethical judgment on utilitarian choice derived from IH: Per scenario, the participants made ethical judgments about the utilitarian choice derived from IH to save five individuals at the expense of killing one by choosing “right” or “wrong.”

They also decided whether or not their choice would affect their life or death for each scenario by selecting “yes” or “no.” This task was intended to check whether the participants correctly understood each context regarding the third- and concerned-party dilemmas.

Empathic concern: After performing the tasks for all scenarios, the participants rated seven items on empathic concern, such as “I often have tender, concerned feelings for people less fortunate than me,” using a five-point scale ranging from 1 (*not agree at all*) to 5 (*agree very much*). These items were the only ones related to empathic concern out of the 28 items of the Japanese version [24] of the Interpersonal Reactivity Index [18,19].

Additionally, the participants’ understanding about the judge’s self-involvement (third-/concerned-party dilemma) was assessed using the question “Would your own life be affected by your decision in this situation?” in each of the four scenarios. They were instructed to respond with “Yes (my life or death depends on my judgment)” or “No (my life or death does not depend on my judgment).” Participants who answered “Yes” to one of the two third-party dilemma scenarios or “No” to one of the two concerned-party dilemma scenarios were deemed to have failed to accurately understand the scenario structure. In addition, after completing the tasks, the participants were instructed to respond with “Yes/No” to the question “Did you answer honestly and discreetly?” to check the sincerity of their responses.

Ethical approval for the experiments in this study was obtained in accordance with the regulations of the Japanese Psychological Association of the first author’s institution (approval number: 2024−01) and by the Ethical Review Committee for Behavioral Research Involving Human Subjects in Kwansei-Gakuin University, Japan.

### Results

Out of 1,300 respondents, 9 were excluded: those who responded “yes (my life or death depends on my judgment)” to one of the two types of third-party dilemmas (i.e., trolley and footbridge) or “no (my life does not depend on my judgment)” to one of the two types of the concerned-party dilemma (department store and cemetery) in the aforementioned self-involvement check items. We also excluded 93 participants who responded “no” to the above question to check the sincerity of their responses. The final number of valid responses was *n* = 1,135 (M = 543, F = 589, unknown = 3, age = 47.1 years, *SD* = 15.7 years).

#### Ethical judgment and empathetic concern per scenario.

In the trolley scenario (third-party/impersonal), 576 (50.7%) and 559 (49.3%) participants indicated that the utilitarian choice derived from IH was ethically “right” and “wrong,” respectively. A significant bias in sex ratio was not observed between these two groups (*χ*^2^ = 1.6, *df* = 1, *p* = 0.21, *φ* = 0.04). The mean score for empathic concern (average of 7 items, α coefficient = 0.80) was M = 3.20 (*SD* = 0.61) in the former group and M = 3.25 (SD = 0.66) in the latter group. A linear mixed-effect model with fixed effects for ethical judgment (approval or disapproval) was applied to the means of empathic concern. The results demonstrated that the fixed effect of ethical judgment was not significant in this scenario (*F*(1,1123) = 1.63, *p* = 0.20, *SE* = 0.04; 95% confidence interval [CI]: −0.03 to 0.12, AIC = 2193.17, BIC = 2208.25). A post-hoc power analysis on effect size (Cohen’s *d* = 0.08) obtained from the *t*-test results between two groups revealed a power (1 − β) of 0.27, which was less than the standard value of 0.8.

In the footbridge scenario (third-party/personal), 238 (21.0%) and 897 (79.0%) participants responded that the utilitarian choice derived from IH was ethically “right” and “wrong,” respectively. No significant bias in sex ratio was observed between them (*χ*^2^ = 1.1, *df* = 1, *p* = 0.31, *φ* = 0.03). Empathetic concern was *M* = 3.10 (*SD* = 0.54) for the former and *M* = 3.25 (*SD* = 0.66) for the latter. The results of the GLMM analysis with ethical judgment as a fixed-effect factor indicated that the fixed effect of ethical judgment was significant (*F*(1, 1123) = 11.26, *p* < 0.001, *SE* = 0.05; 95% CI: 0.07 to 0.25, AIC = 2183.19, BIC = 2198.26). A post-hoc power analysis using the effect size (Cohen’s *d* = 0.25) calculated from a two-group *t*-*t*est indicated a power (1 − β) of 0.99 (>0.8).

In the department store scenario (concerned-party/impersonal), 716 (63.1%) and 419 (36.9%) participants reported that the utilitarian choice derived from IH was ethically “right” and “wrong,” respectively. The study noted no significant bias in sex ratio between them (*χ*^2^ = 3.7, *df* = 1, *p* = 0.06, *φ* = 0.06). Empathic concern was *M* = 3.23 (*SD* = 0.63) for the former and *M* = 3.21 (*SD* = 0.65) for the latter. The GLMM analysis with ethical judgment as a fixed-effect factor did not reveal a significant fixed effect (*F*(1,1123) = 1.63, *p* = 0.20, *SE* = 0.04, 95% CI: −0.10 to 0.06, AIC = 2194.41, BIC = 2209.48). Post-hoc analysis using the effect size (Cohen’s *d* = 0.04) from the *t*-test revealed a power (1 − β) of 0.10 (<0.8).

In the cemetery scenario (concerned-party/personal), 370 (32.6%) and 765 (67.4%) participants indicated that the utilitarian choice derived from IH was ethically “right” and “wrong,” respectively. The sex ratio was not significantly biased between the two groups (*χ*^2^ = 3.6, *df* = 1, *p* = 0.07, *φ* = 0.06). Empathetic concern was *M* = 3.12 (*SD* = 0.63) for the former group and *M* = 3.27 (*SD* = 0.63) for the latter. Conducting GLMM analysis with ethical judgment as a fixed-effect factor revealed a significant fixed effect (*F*(1,1123) = 14.67, *p* < 0.001, *SE* = 0.04, 95% CI: 0.08 to 0.23, AIC = 2180.10, BIC = 2195.17). Post-hoc analysis using the effect size (Cohen’s *d* = 0.24) from the *t*-*t*est indicated a power (1 − β) of 0.97 (>0.8).

**Fig 1:**
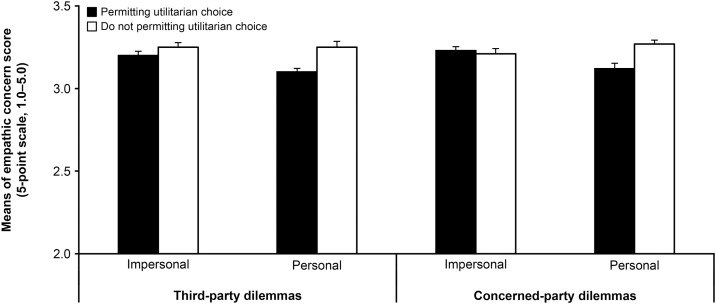
Means of empathic concern for participants depending on their ethical judgments in the third- and concerned-party dilemma.

Fig 1 presents the mean values of empathic concern under the four types of scenarios. GLMM analysis was conducted on the ethical judgments in the four abovementioned dilemmas, with directness of harm (impersonal or personal) and judge’s self-involvement (third-/concerned-party) as the fixed-effect factors. The result indicated that both fixed effects were significant (directness of harm: *F*(1, 3402) = 726.39, *p* < 0.001, *SE* = 0.02, 95% CI: 0.27 to 0.34; judge’s self-involvement: *F*(1, 3402) = 114.87, *p* < 0.001, *SE* = 0.02, 95% CI: −0.15 to −0.09), while the interaction between them was not significant (*F*(1, 3402) = 0.10, *p* = 0.75, *SE* = 0.02, 95% CI: 0.07 to 0.13). Approval of utilitarian judgment in the impersonal scenario was significantly higher than that in the personal scenario for the third- and concerned-party dilemmas: third-party dilemma: 50.6% for the trolley (impersonal) versus 21.0% for the footbridge (personal); concerned-party dilemma: 63.1% for the department store (impersonal) versus 32.6% for the cemetery (personal). At the same time, these results demonstrated that approval of utilitarian judgment was higher for the concerned-party dilemma than for the third-party dilemma, regardless of whether the scenario involved impersonal or personal harm.

#### Effect of empathetic concern on ethical judgment.

To identify the relationship between ethical judgment as the target and empathic concern as a fixed-effect factor, we applied a logistic regression analysis model with GLMM. The fixed effects of the directness of harm and judge’s self-involvement, as well as their interaction with empathic concern, were also modeled to analyze the effects of empathic concern across different types of vignettes (impersonal/personal and third-/concerned-party; Table 1). The resulting effects of empathic concern and the interaction between empathic concern and the directness of the harm were significant. The estimation of the simple slope of empathetic concern in terms of interaction revealed a tendency for utilitarian judgments derived from IH to be suppressed as empathetic concern increased in personal scenarios (OR = 1.45, *p* = 0.00 with 95% CI: 1.15 to 1.81). However, no such significant effect of empathetic concern was observed in impersonal scenarios (OR = 0.96, *p* = 0.70 with 95% CI: 0.77 to 1.20). Empathetic concern was shown to have a suppressive effect on utilitarian judgments derived from IH only in personal scenarios across both the third- and concerned-party dilemmas.

**Table 1:** Effects of empathic concern on ethical judgment in Exp.1 (results of a generalized linear mixed logistic model).

	*p*	OR	95% CI
Intercept	.07	0.46	[0.20; 1.06]
Gender	.03	0.76	[0.59; 0.98]
Age	<.001	1.02	[1.01; 1.03]
Empathic Concern	.00	1.42	[1.11; 1.81]
Directness of harm	.14	0.59	[0.29; 1.18]
Self-involvement	.09	1.78	[0.91; 3.47]
Empathic Concern* Directness of harm	<.001	0.65	[0.52; 0.81]
Empathic Concern* Self-involvement	.33	1.11	[0.90; 1.37]
Empathic Concern* Directness of harm* Self-involvement	.28	0.96	[0.89; 1.04]
Intercept (SD)	<.001	3.35	[2.91; 3.85]

Note: Ethical judgments were coded as “Nonutilitarian” = 1 and “Utilitarian” = 2. Gender was coded as male = 1 and female = 2. Directness of harm was coded as impersonal = 1 and personal = 2. Self-involvement was coded as third-party = 1 and concerned-party = 2.

To investigate more accurately the relationship between ethical judgment and empathetic concern, the participants were categorized in accordance with a previous study [20]:

(1) UTILITARIAN (UTIL): participants that ethically permit utilitarian choices derived from IH in the impersonal and personal scenarios in the third- and concerned-party dilemmas, respectively;
(2) NONUTILITARIAN (NON-UTIL): participants who do not permit utilitarian choices in the impersonal or personal scenarios in the third- and concerned-party dilemmas, respectively;
(3) MAJORITY: participants who permit utilitarian choices derived from IH in the impersonal scenarios but not the personal scenarios in the third- and concerned-party dilemmas, respectively;
(4) OUTLIER: participants with nonutilitarian and utilitarian ethical judgments derived from IH in the impersonal and personal scenarios in the third- and concerned-party dilemmas, respectively.

Table 2 depicts the number and attributes of each UTIL, NON-UTIL, MAJORITY, and OUTLIER and the means for empathic concern in the third- and concerned-party dilemmas.

**Table 2:** Attributes and mean values (SD) of empathic concern for participants depending on their ethical judgments in the third- and concerned-party dilemma.

	Third-party Dilemmas				Concerned-party Dilemmas			
	(Trolley and Footbridge)				(Department store and Cemetery)			
	UTIL	NON-UTIL	MAJORITY	OUTLIER	UTIL	NON-UTIL	MAJORITY	OUTLIER
	*n* = 197	*n* = 518	*n* = 379	*n* = 41	*n* = 318	*n* = 367	*n* = 398	*n* = 52
**Age (*SD*)**	**42.83 (15.08)**	**49.05 (15.36)**	**47.40 (16.25)**	**39.78 (13.13)**	**44.11 (15.58)**	**47.90 (15.40)**	**49.49 (15.76)**	**41.10 (15.00)**
**Gender (M:F)**	**102:94**	**238:279**	**184:194**	**19:22**	**166:151**	**159:207**	**192:205**	**26:26**
**Empathic concern (*SD*)**	**3.07 (0.54)**	**3.25 (0.67)**	**3.26 (0.64)**	**3.23 (0.55)**	**3.12 (0.64)**	**3.22 (0.66)**	**3.32 (0.61)**	**3.10 (0.61)**

The GLMM analysis was conducted on the values for empathic concern in each of the third- and concerned-party dilemmas, with the four-group classification as a fixed-effect factor and gender and age as random-effect factors. The results demonstrated that the fixed effect of the four-group classification was significant for the third- (*F*(3, 1118) = 2.79, *p* = 0.04, *SE* = 0.05, 95% CI: 0.01 to 0.22, AIC = 2123.02, BIC = 2143.09) and concerned-party dilemmas (*F*(1, 1118) = 3.57, *p* = 0.01, *SE* = 0.05, 95% CI: 0.06 to 0.24, AIC = 2121.14, BIC = 2141.22). As a result of multiple comparisons using the Holm method, empathetic concern in UTIL was lower than that in MAJORITY in both the third- and the concerned-party dilemmas (third-party dilemma: *SE* = 0.06, *p* = 0.04, with a 95% CI of −0.30 to −0.01; concerned-party dilemma: *SE* = 0.05, *p* = 0.01, with a 95% CI of −0.27 to −0.02). Accordingly, the findings revealed that those who consistently permit utilitarian choices derived from IH in the impersonal and personal scenarios in the third- or concerned-party dilemma tend to display less empathetic concern than those who make other judgments.

### Discussion

Compared with the third-party dilemma, a higher percentage in the concerned-party dilemma ethically permitted utilitarian choices which led to the self-preservation of the judge in both the impersonal and personal scenarios. This result supports Hypothesis 1a and implies that the judge confronted with a concerned-party dilemma are likely to make a judgment in accordance with the maximin principle and attempt to avoid the maximum disadvantage to themselves (their death).

Conversely, compared with impersonal scenarios, ethical permissiveness for utilitarian choices derived from IH was lower in personal scenarios, which was consistent across the third- and concerned-party dilemmas. Alternatively, results that are in line with those of previous studies (i.e., utilitarian choice is reduced in personal scenarios) were consistent for the third- and concerned-party dilemmas, which supports Hypothesis 1b. This finding indicates that the suggestion in previous studies about the emotional process preventing utilitarian choice derived from controllable processes in moral judgment is also available for the third- and concerned-party dilemmas.

Furthermore, the empathic concern of individuals that permit utilitarian choices derived from IH was lower at only personal scenarios in the third- and concerned-party dilemmas. In addition, in either the third- or concerned-party dilemma, those who consistently permitted utilitarian choices across the impersonal and personal scenarios displayed less empathetic concern than those making other judgments. The observed relationship between empathic concern and ethical judgment in the third-party dilemma is consistent with previous research [20]. The same result was confirmed in the concerned-party dilemma in this study. The results of the logistic regression analysis also demonstrated that empathic concern was a negative determinant of utilitarian ethical judgment derived from IH in only personal scenarios even in the third- and concerned-party dilemmas. These results clearly support Hypotheses 1c: those that permit utilitarian choices derived from IH in personal scenarios exhibit less empathic concern regardless of the type of dilemma.

Experiment 1 illustrated the effect of empathic concern, which consistently restrains utilitarian judgment derived from IH in third- and concerned-party dilemmas with the possibility that people make rational decisions to avoid their maximum disadvantage in the concerned-party dilemma. While the previous study [20] determined a relationship between ethical judgment and empathic concern without categorizing third- and concerned-party dilemmas, the present study, which systematically categorized these two types of dilemma, pointed to particular judgments in the concerned-party dilemma and consistency in moral judgment for both types of dilemma.

## Experiment 2

Experiment 2 investigates ethical judgments in Pareto or non-Pareto concerned-party dilemmas in accordance with previous studies [12,13].

In the context of the Pareto dilemma, the persons who are targets of harm are fixed to the maximum disadvantage for themselves (their death in certain scenarios) regardless of the utilitarian or nonutilitarian choice of the judge [12,13]. By contrast, the targets of harm in the non-Pareto dilemma are given the opportunity to avoid the maximum disadvantage (their death) according to the choice of the judge. The four types of dilemmas in Experiment 1 were non-Pareto scenarios for third- and concerned-party dilemmas. An example of a concerned-party dilemma corresponding to Pareto is the crying baby scenario [12]. The judge is asked whether suffocating a crying baby to save many people on the battlefield, including the judge, is right or wrong, and the nonutilitarian choice (not suffocating the baby) leads to the maximum disadvantage (i.e., everyone gets killed, including the judge and the baby, by enemy soldiers). The Paretian considerations of the judges are activated in Pareto scenarios, and utilitarian choices at the expense of the few are more likely permissible (i.e., for many people, harming someone is permissible if the judgment no longer worsens a person’s condition) [12,25,26].

Evidently, the utilitarian choice is less permissible in personal compared with impersonal scenarios even in the Pareto one, which is consistent whether a dilemma affects the life of the judge or does not affect his/her life [12,13]. In other words, consistent with previous studies that used various scenarios, including third-party dilemmas [5,10,11], it is expected that even in a Pareto concerned-party dilemma in which the many (including the judge) are saved at the expense of the few (whose deaths are fixed nevertheless), the permissiveness of inflicting harm to the few would be lower in personal scenarios.

However, Moore et al. [13] reported that no difference was observed in personal harm between the Pareto and non-Pareto scenarios in the concerned-party dilemma, but only impersonal harm increased in the Pareto scenario. This result may be due to the influences of controllable processes that drive people to make utilitarian choices in impersonal scenarios and of affective mechanisms that suppress controllable processes in personal scenarios [5,10]. Moore et al. argued that the process of distinguishing between Pareto and non-Pareto scenarios is a rational and deliberative cognitive process and positioned it as a part of a process called Paretian preliminaries, which precede the abovementioned Pareto considerations [13]. Pareto considerations and Paretian preliminaries are controllable and deliberative processes. Given that both are controllable, they are considered to enhance permission for utilitarian choices in Pareto scenarios. In personal scenarios, however, it is expected that affective processes (e.g., empathic concern) interfere with controllable processes, including Pareto considerations, which results in less permission for utilitarian choices even in Pareto scenarios. Gleichgerrcht and Young [20] compared moral judgments in Pareto and personal scenarios (crying baby) and non-Pareto and impersonal scenarios (toxic fumes) [20]. In their experiment, utilitarian choice was less in the former than the latter (15.6% in the former versus 61.7% in the latter), because the former was Pareto as well as personal. Additionally, individuals that made the utilitarian choice in the crying baby (personal) scenario displayed less empathetic concern than those who made the nonutilitarian choice. These results indicate that utilitarian choices are less likely to be made due to the effects of empathic concern even in a Pareto but personal scenario. However, the crying baby and toxic fumes scenarios are distinct from each other not only in terms of personal/impersonal but also in terms of Pareto/non-Pareto scenario, and the former and latter are concerned- and third-party dilemmas, respectively. Given the direct comparison of different scenarios across several factors, the experiment by Gleichgerrcht and Young can hardly be described as yielding a systematic view of the interaction between Pareto considerations and empathic concern.

In Experiment 2, the current study prepared four types of scenarios: Pareto and non-Pareto as well as personal and impersonal scenarios in the concerned-party dilemma. It tests the hypothesis that empathic concern suppresses direct personal harm to others in a Pareto but personal scenario. Experiment 2 is intended to test the following hypotheses. None of the hypotheses were pre-registered; however, they were developed on the basis of previous theoretical deductions. All manipulations in Experiment 2 and subsequent analyses were performed based on these hypotheses.

Hypothesis 2a: Ethical permission of the utilitarian choice derived from IH would be lower in the personal scenario in Pareto or non-Pareto scenarios.

Hypothesis 2b: Ethical permission of the utilitarian choice derived from IH would be higher in Pareto than in non-Pareto scenarios only in impersonal scenarios.

Hypothesis 2c: In the Pareto and non-Pareto scenarios, the suppressive effects of empathic concern for the utilitarian choice derived from IH would be found only in personal scenarios.

### Participants

Participants were recruited separately from those in Experiment 1. They were native Japanese speakers aged 18 years or older who registered with a research company, responded to the online survey. Data collection began on 10 December 2024 and was completed within one week, on 16 December 2024. The total number of respondents was 1,300. They also provided written informed consent on a web-based form. The number and attributes of valid respondents are described in a later section.

### Materials and methods

Four scenarios (i.e., department store, cemetery, hibernation, and lifeboat) were prepared according to two (within-participant) factors: tradeoff structure (2: non-Pareto or Pareto) × directness of harm (2: impersonal or personal) in moral dilemmas. The department store (non-Pareto/impersonal) and cemetery (non-Pareto/personal) scenarios are identical to those in Experiment 1. The hibernation (Pareto/impersonal) scenario consisted of a utilitarian choice derived from IH to cut electric power to an artificial hibernation unit for one person to save five people, including the judge, on a spaceship that ran out of energy due to an accident. The nonutilitarian choice is not to cut the power (resulting in the deaths of all six persons, including the judge). The utilitarian choice derived from IH in the lifeboat (Pareto/personal) scenario was to push one unconscious person overboard to save five persons, including the judge, from a sinking lifeboat occupied by six persons, while the nonutilitarian choice was not to push the one person overboard (resulting in the sinking of the boat and the death of all six persons, including the judge).

In the non-Pareto scenario (department store and cemetery scenarios), the utilitarian or nonutilitarian choice of the judges will always save five or one person, respectively, while one person was fixed to die in the Pareto scenario (hibernation and lifeboat scenarios) regardless of the choice of the judge. The impersonal scenario depicts a situation in which the choice to save five persons indirectly caused harm to one person, while the personal scenario was a situation in which the choice to save five persons required directly and intentionally harming one person. Equivalence of outcomes in terms of number and severity was maintained throughout the four scenarios in the form of one death and five saves. All scenarios were a concerned-party dilemma, and the judge was always assigned the position as one of the five persons.

### Procedures

The same procedure in Experiment 1 was employed. The participants were randomly presented with four scenarios on the web, namely, department store, cemetery, hibernation, and lifeboat. They made an ethical judgment (“right” or “wrong”) about a utilitarian choice derived from IH (a choice to victimize one person to save five, including the participant) per scenario and responded to seven items on empathic concern (rated using a five-point scale) after completing the tasks for all scenarios. Furthermore, the study checked for the participants’ understanding of the tradeoff structure of the four scenarios (non-Pareto or Pareto) immediately after reading each scenario by selecting “depending on the judge’s choice, all six, including five and one persons, will not be saved” or “either five or one person will certainly be saved.” For this check item, based on our established criterion, the respondents were deemed to have misunderstood the tradeoff structure of the scenarios if they selected “all six, including five and one persons, will not be saved” in one of the two non-Pareto scenarios (department store and cemetery) or if they selected “either five or one person will certainly be saved” in one of the two Pareto scenarios (hibernation and lifeboat). In addition, the participants were instructed to indicate “Yes/No” to the question regarding the sincerity of their responses after completing all tasks, similar to Experiment 1 (“Did you answer honestly and discreetly?”).

### Results

Out of 1,300 participants, we excluded a total of 348 respondents who selected “all six, including five and one persons, will not be saved” to the abovementioned item on the tradeoff structure in either of the two non-Pareto scenarios (department store and cemetery) or “either five or one person will certainly be saved” in either of the two Pareto scenarios (hibernation and lifeboat). The Paretian preliminaries, which cognitively distinguish between *Pareto* and *non-Pareto* scenarios, can be assumed to involve controllable processes [13]. The present experiment also should have required the activation of controllable processes to enable the participants to accurately understand the “Pareto” and “non-Pareto” scenarios. However, the actuation of this process is thought to impose a considerable cognitive workload, which likely hindered participants’ full comprehension of the tradeoff structure, thus leading to an increase in the number of eliminations (the abovementioned 348 participants). Moreover, we excluded 25 respondents who responded “No” to the question, “Did you answer honestly and thoughtfully?,” which pertains to their sincerity. These consecutive eliminations were conducted following the criteria established prior to the experiment, and selection effects were avoided. The final number of valid responses was *n* = 927 (M = 478, F = 446, unknown = 3, age = 45.3 years, *SD* = 14.8).

#### Ethical judgment and empathetic concern per scenario.

For the department store scenario (non-Pareto/impersonal), 605 (65.3%) and 321 (34.7%) participants indicated that the utilitarian choice derived from IH was ethically “right” and “wrong,” respectively. Between them, the study found a significant bias in sex ratio (*χ*^2^ = 7.08, *df* = 1, *p* = 0.01, *φ* = 0.09). The score of empathic concern (mean of 7 items, α coefficient = 0.85) was *M* = 3.17 (*SD* = 0.69) for the former and *M* = 3.25 (*SD* = 0.68) for the latter. A GLMM analysis with ethical judgment (permission or prohibition) as the fixed-effect factor was conducted on empathetic concern. The results illustrated that the fixed effect was not significant in this scenario (*F*(1, 920) = 2.96, *p* = 0.09, *SE* = 0.05, 95% CI: −0.01 to 0.18, AIC = 1935.49, BIC = 1949.97). Post-hoc analysis on effect size (Cohen’s *d* = 0.12) derived from a two-group *t*-test yielded a power (1 − β) of 0.41 (<0.8).

In the cemetery (non-Pareto/personal) scenario, 432 (46.6%) and 495 (53.4%) participants indicated that the utilitarian choice derived from IH was ethically “right” and “wrong,” respectively. The study noted a significant bias in sex ratio between them (*χ*^2^ = 5.06, *df* = 1, *p* = 0.03, *φ* = 0.07). Empathic concern was *M* = 3.11 (*SD* = 0.69) for the former and *M* = 3.29 (*SD* = 0.67) for the latter. The GLMM results indicated that the fixed effect of ethical judgment was significant (*F*(1, 921) = 16.03, *p* < 0.001, *SE* = 0.05, 95% CI: 0.09 to 0.27, AIC = 1924.02, BIC = 1938.50). Post-hoc analysis with *t*-*t*est effect size (Cohen’s *d* = 0.26) revealed a power (1 − β) of 0.98 (>0.8).

In the hibernation scenario (Pareto/impersonal), 559 (60.4%) and 367 (39.6%) indicated that the utilitarian choice derived from IH was ethically “right” and “wrong,” respectively. A significant bias in sex ratio was observed between both groups (*χ*^2^ = 17.48, *df* = 1, *p* < 0.001, *φ* = 0.14). Empathic concern was *M* = 3.12 (*SD* = 0.70) for the former and *M* = 3.33 (*SD* = 0.64) for the latter. The result of the GLMM analysis indicated that the fixed effect of ethical judgment was significant (*F*(1, 920) = 20.37, *p* < 0.001, *SE* = 0.05, 95% CI: 0.12 to 0.30, AIC = 1917.38, BIC = 1931.84). Post-hoc analysis on *t*-tes*t* effect size (Cohen’s *d* = 0.30) yielded a power (1 − β) of 0.99 (>0.8).

In the lifeboat scenario (Pareto/personal), 460 (49.7%) and 466 (50.3%) participants responded that the utilitarian choice derived from IH was ethically “right” and “wrong,” respectively, with a significant bias in sex ratio between them (*χ*^2^ = 6.14, *df* = 1, *p* < 0.01, *φ* = 0.08). Empathic concern was *M* = 3.12 (*SD* = 0.72) for the former and *M* = 3.28 (*SD* = 0.65) for the latter. The results of the GLMM analysis highlighted that the fixed effect of ethical judgment was significant (*F*(1, 920) = 11.71, *p* < 0.001, *SE* = 0.05, 95% CI: 0.07 to 0.24, AIC = 1926.68, BIC = 1941.16). Post-hoc analysis using the *t*-tes*t* effect size (Cohen’s *d* = 0.23) revealed a power (1 − β) of 0.94 (>0.8).

**Fig 2:**
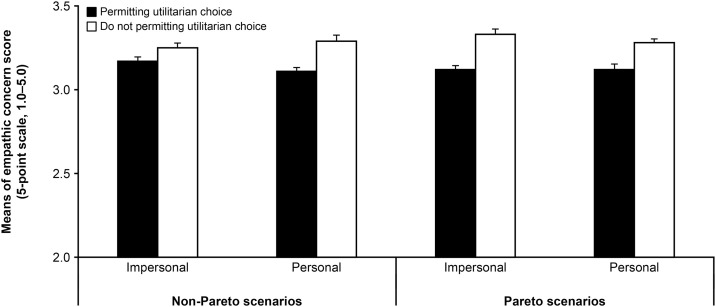
Means of empathic concern for participants depending on their ethical judgments in the non-Pareto and Pareto scenario.

The mean values for empathic concern in each of the four scenarios are shown in Fig 2. The levels of empathic concern are lower among individuals who ethically permit utilitarian choice in three scenarios without the non-Pareto and impersonal scenario.

The GLMM analysis with directness of harm (impersonal or personal) and tradeoff structure (non-Pareto or Pareto) for fixed-effect factors was conducted on ethical judgment in the four abovementioned dilemmas. The result emphasized that the fixed effect of harm was significant (*F*(1, 2774.73) = 159.12, *p* < 0.001, *SE* = 0.02, 95% CI: 0.07 to 0.13), but the specific effect of the tradeoff structure was not significant (*F*(1, 2774.73) = 0.71, *p* = 0.40, *SE* = 0.02, 95% CI: −0.06 to 0.00). Conversely, the interaction was significant (*F*(1, 2774.73) = 12.16, *p* < 0.001, *SE* = 0.02, 95% CI: 0.04 to 0.13). Comparing between impersonal scenarios (department store [non-Pareto] and hibernation [Pareto]), the rate of utilitarian judgment derived from IH was higher in the former (65.3%) than in the latter (60.4%). Conversely, comparing between the cemetery (non-Pareto) and lifeboat (Pareto) scenarios, which are personal scenarios, the rate of utilitarian judgment was higher in the latter (49.7%) than in the former (46.6%). The tendency for utilitarian judgment derived from IH to increase in the impersonal scenario compared with the personal scenario was consistently observed in the non-Pareto and Pareto cases.

Thus, we found differences in empathic concern according to the ethical judgment for the utilitarian choice derived from IH in all scenarios, except for the department store scenario. In addition, we observed that permissions for utilitarian choices derived from IH were higher for non-Pareto than for Pareto in impersonal scenarios, and they consistently decreased more in personal compared with impersonal scenarios for non-Pareto and Pareto.

#### Effect of empathetic concern on ethical judgment.

To examine the influence of empathetic concern across different scenario types (non-Pareto/Pareto, impersonal/personal), the GLMM logistic model was applied targeting ethical judgments and fixed effects for empathetic concern, the directness of harm, the trade-off structure, and their interactions. The fixed effects of empathetic concern and directness of harm were significant, along with a significant interaction between empathetic concern, directness of harm, and the trade-off structure (Table 3). The simple slope of empathic concern was not significant only for the department store scenario (non-Pareto/impersonal; OR = 1.19, *p* = 0.10 with a 95% CI of 0.97 to 1.47). It was significant in the cemetery (non-Pareto/personal; OR = 1.42, *p* < 0.001 with a 95% CI of 1.16 to 1.74), hibernation (Pareto/impersonal; OR = 1.46, *p* < 0.001 with a 95% CI of 1.18 to 1.80), and lifeboat scenarios (Pareto/personal; OR = 1.30, *p* = 0.01 with a 95% CI of 1.07 to 1.59). While the suppressive effect of empathic concern on utilitarian judgments derived from IH was observed only in a personal for non-Pareto scenario, it was observed in both personal and impersonal for Pareto scenarios.

**Table 3:** Effects of empathic concern on ethical judgments in Exp.2 (results of a generalized linear mixed logistic model).

	*p*	OR	95% CI
Intercept	<.001	0.19	[0.07; 0.53]
Gender	<.001	0.56	[0.40; 0.77]
Age	.38	1.01	[0.99; 1.02]
Empathic Concern	<.001	1.75	[1.31; 2.33]
Directness of harm	.01	0.41	[0.21; 0.84]
Tradeoff structure	1.00	1.78	[0.90; 3.53]
Empathic Concern* Directness of harm	.78	1.03	[0.83; 1.28]
Empathic Concern* Tradeoff structure	.33	0.90	[0.73; 1.11]
Empathic Concern* Directness of harm* Tradeoff structure	<.001	0.81	[0.75; 0.89]
Intercept (SD)	<.001	4.87	[4.25; 5.58]

Note: Ethical judgments were coded as “Nonutilitarian” = 1 and “Utilitarian” = 2. Gender was coded as male = 1 and female = 2. Directness of harm was coded as impersonal = 1 and personal = 2. Tradeoff structure was coded as non-Pareto = 1 and Pareto = 2.

Consistent with Experiment 1, the participants were classified under four types for each of the non-Pareto and Pareto scenarios according to ethical judgment, namely, UTILITARIAN (UTIL), NONUTILITARIAN (NON-UTIL), MAJORITY, and OUTLIER. Table 4 presents the number, attributes, and means of sympathetic concern for the four types in non-Pareto and Pareto scenarios, respectively.

**Table 4:** Attributes and mean values (SD) of empathic concern for participants depending on their ethical judgments in the non-Pareto and Pareto scenario.

	Non-Pareto Dilemmas				Pareto Dilemmas			
	(Department store and Cemetery)				(Hibernation and Lifeboat)			
	UTIL	NON-UTIL	MAJORITY	OUTLIER	UTIL	NON-UTIL	MAJORITY	OUTLIER
	*n* = 379	*n* = 269	*n* = 226	*n* = 52	*n* = 397	*n* = 304	*n* = 161	*n* = 63
**Age (*SD*)**	**44.96 (14.83)**	**45.33 (14.78)**	**46.90 (14.95)**	**41.44 (12.98)**	**44.56 (14.79)**	**47.66 (14.34)**	**44.24 (14.80)**	**42.03 (15.59)**
**Gender (M:F)**	**214:164**	**121:148**	**117:107**	**26:26**	**228:167**	**131:173**	**91:69**	**28:35**
**Empathic concern (*SD*)**	**3.11 (0.69)**	**3.29 (0.67)**	**3.28 (0.67)**	**3.09 (0.71)**	**3.11 (0.73)**	**3.34 (0.65)**	**3.16 (0.62)**	**3.24 (0.58)**

The GLMM analysis was conducted on the values of empathic concern, with the abovementioned four-group classification as a fixed-effect factor and gender and age as random-effect factors for the non-Pareto and Pareto scenarios. The result indicated that only the fixed effect of the four-group classification was significant for the non-Pareto (*F*(3, 918) = 4.08, *p* = 0.01, AIC = 1871.95, BIC = 1891.24) and Pareto (*F*(3, 916) = 4.56, *p* = 0.00, AIC = 1867.09, BIC = 1886.37) scenarios. The results of multiple comparisons using the Holm method revealed that empathic concern in UTIL was lower than in NON-UTIL in the non-Pareto (*SE* = 0.05, *p* = 0.02, 95% CI: −0.30 to −0.02) and Pareto (*SE* = 0.05, *p* = 0.00, 95% CI: −0.32 to −0.05) scenarios. People who consistently permit utilitarian judgment derived from IH in the impersonal and personal scenarios exhibited lower levels of empathic concern than those who consistently make nonutilitarian judgment in the non-Pareto or Pareto scenarios.

### Discussion

Comparing between the impersonal and personal scenarios, ethical permission for utilitarian choices derived from IH was consistently lower in the latter in non-Pareto and Pareto scenarios, which supports Hypothesis 2a. Previous studies repeatedly point out the suppression of personal harm relative to impersonal harm [5,10,11], which Experiment 2 also confirmed in the Pareto concerned-party dilemma.

Comparing between non-Pareto (department store) and Pareto (hibernation) impersonal scenarios, the former was more ethically permissive of utilitarian choices derived from IH than the latter. This finding is inconsistent with a previous study [13] that reported that utilitarian choices are more likely to be permitted in impersonal (Pareto) scenarios. The results of the logistic regression analysis implied that while empathic concern suppressed ethical permission for utilitarian choices derived from IH in the Pareto/impersonal (hibernation) scenario, no such suppressive effects were observed in the non-Pareto/impersonal (department store) scenario. In the current scenarios, the target person to harm was a stranger, but only in the Pareto/impersonal (hibernation) scenario. The description of members in the same spaceship could have led the judge to interpret it as an acquainted coworker. This aspect may have resulted in the judges feeling familiar with the target person to harm and evoked situational empathy [27,28] in the current context.

Alternatively, in the personal scenarios, the study found no difference in ethical permission for utilitarian choices derived from IH between the non-Pareto (cemetery) and Pareto (lifeboat) scenarios. This result is consistent with a previous study [13]. Furthermore, the results of logistic regression analysis pointed to a suppressive effect of empathic concern on permission for utilitarian choices derived from IH in the non-Pareto and Pareto (personal) scenarios, which partially support Hypothesis 2b. Paretian consideration and Paretian preliminaries are regarded as rational and deliberative processes [12,13]; however, intuitive processes are likely to suppress these deliberative processes in personal scenarios, even in the Pareto concerned-party dilemma as well as non-Pareto scenarios.

In the case of non-Pareto scenarios, the participants that permit utilitarian choices derived from IH exhibited less empathic concern only in the personal (cemetery) scenario. This difference between the two groups was not observed in the impersonal (department store) scenario. In addition, based on the results of the logistic regression analysis, empathetic concern exerted a suppressive effect on utilitarian judgment derived from IH in personal scenarios. Furthermore, those who consistently permitted utilitarian choices across the impersonal and personal scenarios displayed less empathic concern. The results obtained from the non-Pareto scenarios are in line with that of previous study [20] and are consistent with the results of Experiment 1.

In the Pareto case, those who permitted utilitarian choices derived from IH displayed less empathic concern in the impersonal (hibernation) and personal (lifeboat) scenarios. Logistic regression analysis also indicated that empathic concern suppressed utilitarian judgment derived from IH in the impersonal and personal scenarios. Consistent with the abovementioned non-Pareto case, we observed that participants who consistently permitted utilitarian choices across the impersonal and personal (Pareto) scenarios displayed less empathetic concern. These results partially support Hypothesis 2c and indicate that empathic concern is capable of suppressing harm to others even in concerned-party dilemmas with a Pareto structure.

## General discussion

We examined ethical judgment in each of the third- and concerned-party dilemmas with a focus on empathic concern based on previous studies [18–20]. First, ethical permission for utilitarian choices derived from IH was higher in the concerned-party dilemma than in the third-party dilemma under the impersonal or personal scenarios. Alternatively, empathic concern was consistently a determinant of ethical judgment in third- and concerned-party dilemmas, especially in personal scenarios. Furthermore, in the concerned-party dilemma, we confirmed that empathic concern remains influential in the Pareto scenario that involves Paretian considerations, which is supposedly a deliberative process. These results indicate the particular or common aspects of the impact of each context of the third- and concerned-party dilemmas on moral judgment. The study infers that the reason underlying the higher permission of utilitarian choices derived from IH in concerned-party dilemmas that a controllable process, in which the judge considers his/her interests in accordance with the maximin principle, should be involved only in this kind of dilemmas. In contrast, the observation that empathic concern, as a type of altruistic emotion, suppressed utilitarian judgment in the third- and concerned-party dilemmas—especially in the personal scenario—indicates that the intuitive process that influences moral judgment is common to the two types of dilemmas. These results revealed that certain scenarios among various moral dilemmas arouse the rational decision-making process of individuals, which aims to avoid their own worst-off. Conversely, empathic concern may also interfere with the rational process even in these types of scenarios.

To classify various forms of moral dilemmas, this study proposed two criteria: third-/concerned-party and non-Pareto/Pareto. Although Moore et al. [8,13] examined self-dilemmas with regard to the definition and categorization of the third- and concerned-party dilemmas proposed in this study, a systematic investigation on moral judgment remains lacking. Similarly, few studies have classified moral dilemma scenarios based on criteria for non-Pareto/Pareto definitions with a systematic examination of the criterion [12,13]. Therefore, previous studies [5,10,11] combined two types of scenarios in which the self-interest of the judge was excluded or included in each of the impersonal or personal scenarios. The context of a third-party dilemma does not involve a controllable process in which people estimate their maximum disadvantage in making ethical decisions (i.e., a decision-making process along with the maximin principle). However, this process may impact ethical judgments in the concerned-party dilemma. This notion indicates that these two types of dilemmas should not be included under the same category, because of their different processes in making a judgment, as well as the distinction between impersonal and personal dilemmas. The categorization of third-/concerned-party and non-Pareto/Pareto dilemmas provides another perspective for examining the role of moral and rational processes, which are supposedly independent of each other, in the process of making moral judgments.

As an example, studies that use moral dilemmas such as the trolley problem have been criticized as unrealistic and poorly plausible owing to the scenarios they have adopted [29]. A potential approach for overcoming these concerns is to devise more realistic scenarios based on actual cases in society [30,31]. The 24 dilemmas developed by Gawronski et al. [30] and the 48 dilemmas proposed by Körner et al. [31] include scenarios based on actual cases: debates on the ethics of disconnecting life support to obtain organs for transplantation, conducting torture to obtain information on abducted children in Canada, and discussing the Ebola infection of Dr. Kent Brantly in Liberia and his repatriation to the United States for treatment. However, even these realistic scenarios could be adopted while the distinction between third-/concerned-party remains ambiguous. The Dr. Kent Brantly scenario presents a concerned-party dilemma for individuals who assume that the doctor’s home country is the same as their own (the scenario does not explicitly state that the actual incident occurred in the United States), whereas those who understand that the doctor’s home country is different from their own would perceive it as a third-party dilemma.

Therefore, to standardize or manipulate people’s understanding of the context of the scenario, it is essential to classify various moral dilemmas according to their structure even when they represent unrealistic scenarios, like the trolley problem. The classification and manipulation of moral dilemmas corresponding to the possibility that one’s own judgment may affect not only others but also one’s own interests (third-/concerned-party) or the possibility that one’s own judgment may improve the interests of a minority (non-Pareto/Pareto) are key to independently examining the effects of moral and rational processes on moral judgments. The crucial point of this study was to define the dilemmas in which rational processes may or may not reliably interfere in people’s moral judgments and propose methods for manipulating such dilemmas. Such manipulations must be accompanied by checks to confirm that participants have correctly understood the structure of each scenario. The introduction of an operation check in this study resulted in many exclusions, which will be discussed later as a theoretical limitation.

At this stage, a question emerges in terms of the extent to which self-interested moral dilemmas, such as the concerned-party dilemma in our study, are realistic in daily life. Huebner et al. [12] argued that moral dilemmas with the Pareto structure are unfamiliar and are limited to atypical situations such as battlefields or emergencies. Alternatively, Moore et al. [13] mentioned that everyday moral dilemmas typically involve costs and benefits for the judge. An important example of a Pareto concerned-party dilemma confront in everyday life could be various problems in public goods dilemmas with a NIMBY (not in my backyard) structure. NIMBY refers to public facilities (e.g., nuclear facilities and waste disposal plants) that provide public benefits to the majority in society but impose various risks or disadvantages to residents in the area where they are located and people’s reactions to these facilities [32–34]. Needless to say, many contexts related to NIMBY-type public facilities are not life-or-death situations such as on the battlefield or in emergencies in which a person dies for saving the lives of others. Given that the context involves a conflict of interest between the majority in society and residents of the site [35], a structure of moral dilemma that focuses on the rightfulness or wrongfulness of a judgment to save the many at the expense of the few is established. In addition, NIMBY-type public facilities involve the structure of a social dilemma in which the location of such facilities in a neighborhood forces individuals in the worst position; if they reject it, the facility will still not be relocated, and public interest will be undermined consequently [36]. When people judge on the side of the majority in this context, a Pareto concerned-party dilemma is constituted in which saving the minority (i.e., terminating the location of a facility that could result in the worst position for the residents of the site) results in undermining the public interest for all of the majority and minority. Just as empathic concern was a suppressive determinant of utilitarian choices in the Pareto concerned-party dilemma, the current study infers that empathic concern will undermine the public interest of all people in a NIMBY-type public goods dilemma if it drives people to refuse the sacrifices of the few in terms of public goods. Indeed, although the brain regions associated with attention to worst outcomes (right angular gyrus) are involved in NIMBY-type public goods dilemmas, other brain regions (e.g., amygdala) that evoke emotional aversion to utilitarian choices are also involved, which is common among moral dilemmas [37]. In addition, it was demonstrated that ethical judgment in moral dilemmas (e.g., the trolley problem) is related to ethical judgment for utilitarian choices that prioritize the welfare of the majority over the cost of the minority in the public goods dilemma [17].The study expects that a further examination of moral judgment in each of the third- or concerned-party dilemma will lead to an approach for formulating perspectives based on the literature on moral research to address the moral tragedy in the public goods dilemma. The present study is positioned as a first step in these efforts.

## Theoretical limitations

This study aimed to propose a distinction and definition between third- and concerned-party dilemmas for research based on moral dilemmas and to demonstrate common or different points between these two types of dilemmas, primarily from the perspective of the relationship between ethical judgment and empathic concern. While the study achieved this purpose to a certain extent, four theoretical limitations should be noted regarding its implications.

First, it involved numerous data exclusions due to manipulation checks. In Experiments 1 and 2, the participants’ understanding of the third-/concerned-party dilemma and non-Pareto/Pareto dilemma, respectively, was checked. Based on the check, more than 300 participants were excluded in Experiment 2. The vignettes used in this study had not undergone pilot tests to assess the participants’ understanding of their contextual structure. This shortcoming may have contributed to the high exclusion rate in the main experiments. However, prior studies also did not confirm whether participants accurately understood the structure of dilemmas—such as whether they were third- or concerned-party or whether they were non-Pareto or Pareto [12,13]. The fact that the operational check introduced in this study resulted in a high exclusion rate might also suggest that there were procedural deficiencies in previous studies and uncertainty regarding the validity of their reports. The participants’ accurate understanding of the dilemmas is presupposed in examinations of their moral judgment. Thus, future research should undertake such checks. Furthermore, a procedure should be designed to decrease the cognitive load of participants while providing a more accurate understanding of the dilemma structure. Although this study did not include a pilot test, the experiment itself could be regarded as a pilot study, with the potential to inspire improvements in future experimental procedures investigating moral judgments.

Second, although the scenarios were standardized to establish that the target of harm was a stranger to the judge, only one of the Pareto (hibernation) scenarios was described in a manner that enabled it to be perceived as a familiar person. This aspect may have prevented a clear explanation of the results of Experiment 2. At the onset, only one scenario was prepared for each of the combinations of the three classification criteria: third-/concerned-party dilemma, impersonal/personal scenarios, and non-Pareto or Pareto structure; thus, without mentioning that the validity of the results in our study is not so high. Thus, the need also emerges for future studies that aim to reanalyze data in the various scenarios presented by previous scholars [5,11,15] according to the definitions of concerned- and third-party dilemmas. The true significance of the approach for applying the achievements of studies on moral judgment to public goods issues that exhibit the structure of social dilemmas should be exercised thereon.

Third, this study focused on utilitarian IH, whereas IB was not examined. The study only employed a dichotomous scale, asking participants to judge utilitarian choices as either “right” or “wrong.” When investigating the hypotheses based on previous studies, it was necessary to adopt the identical scale as those studies. However, although the ethical judgment derived from the dichotomous scale was inappropriate for capturing IB, it effectively captured IH. The former, IH, is regarded as the negative aspect of utilitarianism, while the latter, IB, is deemed the core and positive aspect of utilitarianism [21]. The common or different points between the third- and concerned-party dilemmas are limited to IH only. To apply these insights to IB, further examination involving the Oxford Utilitarianism Scale, which measures IH and IB separately, is required [21,22].

Finally, inaction bias may have occurred because all sacrificial scenarios involved a certain type of action (e.g., pulling the lever and pushing one person off) as a utilitarian choice, whereas all nonutilitarian choices were inactions (e.g., not pulling the lever and not pushing one person off). Inaction bias refers to the tendency for harm caused by action to be perceived as more severe than the same harm caused by inaction [38,39]. The tendency for people to prefer inaction may increase the rate of nonutilitarian judgment and decrease the rate of utilitarian judgment through pathways distinct from moral judgment for all scenarios. The CNI (Consequences, Norms, and Inaction Versus Action) Model of Moral Dilemma Responses [30,40] asserts that people’s reactions to moral dilemmas are jointly influenced by “a person’s sensitivity to consequences for the greater good (C),” “sensitivity to moral norms of harm and care (N),” and “general preference for inaction versus action (I).” In accordance with the model, two factors may have influenced the nonutilitarian judgment in the trolley problem-type scenarios in the present study: preferences for moral norms (N parameter) and inaction (I parameter). Separating these two impacts is impossible within a paradigm that involves only trolley problem-type scenarios [30]. However, Körner et al. [31] reported that empathic concern was positively correlated with the N parameter in the CNI model, whereas no correlation was observed for the C parameter (sensitivity to consequences) or the I parameter. On the basis of their report, the current results—which indicated a negative correlation between empathic concern and utilitarian choice in the third- and concerned-party dilemmas, but only in the personal scenario—are attributed to sensitivity to moral norms instead of inaction bias. Regardless, the findings of this study, which used only trolley problem-type sacrificial dilemmas, cannot overlook the possibility of intervention from inaction bias. Thus, constructing realistic third- and concerned-party dilemma scenarios based on the CNI model and reexamining the relationship between reactions in these scenarios and empathic concern are potential strategies for enhancing the validity of the present findings.

## Supporting information

S1 Appendix The moral dilemma scenarios selected for Experiments 1 and 2 in this study. (DOCX)
