# Supplementary material for: Third- and concerned-party positions in moral dilemmas: Effect of empathy on ethical judgment
Source: PLoS One. 2026 Jul 30;21(7):e0354169. doi: 10.1371/journal.pone.0354169 (PMC13422854; doi:10.1371/journal.pone.0354169)
Supplement: S1 Appendix — (DOCX) [file pone.0354169.s001.docx]

**S1 Appendix**

**Scenarios**

Trolley (impersonal/third-party/non-Pareto)

When taking a walk on the side of the railroad tracks, you see a trolley that is out of control and speeding toward here. If it continues, then five workers on the track will be run over and killed. Next to the tracks, you see a lever in front of you that switches the tracks. If you pull the lever, then the track will switch, which will place on the spare track and save the five workers. However, one worker is standing on the spare track, and if you pull the lever, then that one worker will die.

Footbridge (personal/third-party/non-Pareto)

You are walking on a footbridge that crosses over the railroad tracks. A runaway trolley is rushing onto five workers on the railroad tracks, which you can see from the footbridge, and they will die if this situation continues. There happens to be a large-bodied stranger right next to you, also overlooking the tracks. If you push this stranger off the footbridge, then the trolley will be derailed as soon as it collides with his large body. The stranger will die, but the five workers will be saved.

Department store (impersonal/concerned-party/non-Pareto)

While shopping in a department store, an earthquake strikes, and you are trapped in the store with four other shoppers who are strangers to you. Toxic fumes from the fire are penetrating your area through the ventilation ducts; if this continues, then you and the four other shoppers will die. If you turn on the ventilation switch on the wall nearby, then the ducts will change over, and you all will be saved. However, in this case, the toxic fumes will flow into the next area, thus killing one other unknown shopper who is trapped there.

Cemetery (personal/concerned-party/non-Pareto)

You are hiking through the woods. You came across an open space where five hikers, all strangers, are camping, and you pitch your tent. However, it is the sacred cemetery of a local clan. The clan elders decreed that you will be executed in accordance with their code. They gave you the clan’s sword and told you that if you stabbed one of the unknown hikers to death using the sword, then you and the four remaining hikers would be released. If you refuse, they will kill you and the four hikers and release one.

Hibernation (impersonal/concerned-party/Pareto)

Six crew members on a spaceship are in artificial hibernation. When you awoke for your shift, the power reactor severely failed. Continuing in this state, the ship will run out of energy, and all six, including you, will not survive. If you turn off the power switch of the unit for one of the hibernating crew, then it will leave just enough energy for the five other members of the crew, including you, barely to survive. Evidently, the one in the unit whose power switch is turned off will die.

Lifeboat (personal/concerned-party/Pareto)

A fire broke out on a cruise ship. The passengers escaped, but six mutual strangers, including you, occupied a lifeboat with a capacity of five, and it was nearly sinking due to overweight. If this continues, the boat will sink, and all six passengers, including you, will drown to death. If you push one severely injured passenger next to you overboard, then the boat will lighten up, and the other five passengers, including you, will be saved. Evidently, the one thrown overboard will shortly die in the sea.
